# Characterization of Oligopeptides in *Solanum lycopersicum* Xylem Exudates

**DOI:** 10.3390/life12040592

**Published:** 2022-04-16

**Authors:** Satoru Okamoto, Azusa Kawasaki, Yumiko Makino

**Affiliations:** 1 Graduate School of Science and Technology, Niigata University, Niigata 950-2181, Japan; azutack@ma.tlp.ne.jp; 2 PRESTO, Japan Science and Technology Agency, Kawaguchi 332-0012, Japan; 3 National Institute for Basic Biology, Okazaki 444-8585, Japan; makino@nibb.ac.jp

**Keywords:** xylem, long-distance, tomato (*Solanum lycopersicum*), peptide, CLV3/ESR-related (CLE), C-TERMINALLY ENCODED PEPTIDE (CEP), CASPARIAN STRIP INTEGRITY FACTOR (CIF)

## Abstract

The xylem is the main pathway for the transport of water and molecules from roots to shoots. To date, it has been reported that secreted oligopeptides mediate root-to-shoot signaling, and some long-distance mobile oligopeptides have been detected in xylem exudates. However, the conservation of a number of oligopeptides and the overall features of peptide fragments contained in xylem exudates are poorly understood. Here, we conducted a comprehensive analysis of small proteins and peptides in tomato (*Solanum lycopersicum*) xylem exudates and characterized the identified peptide fragments. We found that putative secreted proteins were enriched in xylem exudates compared with all proteins in the tomato protein database. We identified seven oligopeptides that showed common features of bioactive oligopeptides, including homologs of CLV3/ESR-related (CLE), C-TERMINALLY ENCODED PEPTIDE (CEP), and CASPARIAN STRIP INTEGRITY FACTOR (CIF) peptides. Furthermore, five of the identified oligopeptides were homologs of the soybean xylem exudate-associated oligopeptides that we previously reported. Our results suggest that oligopeptides in xylem exudates are conserved across plant species and provide insights into not only root-to-shoot signaling but also the maintenance of the xylem conduit.

## 1. Introduction

The xylem is the main pathway for the transport of water and micronutrients from roots to shoots. In addition, for more than three decades, a variety of proteins have been identified from xylem exudates in different plant species [1,2,3,4,5,6,7,8,9]. The vast majority of proteome analyses of xylem exudates have been performed using conventional gel-based purification techniques and have focused on relatively large proteins. To date, some protein families have been commonly found among different plants, such as peroxidases and lipid transfer proteins (LTPs). Some peroxidases are involved in cell wall lignification and defense responses against vascular pathogens [10,11]. On the other hand, one of the LTPs, Defective in induced resistance 1, is suggested to mediate systemic acquired resistance via the phloem through the interaction with glycerol-3-phosphate [12,13]. Although the exact functions of each xylem-associated protein have not been clarified, considering those findings and their conservation in xylem exudates across plant species, these proteins might play fundamental roles in plants.

In plants, it is well known that secreted oligopeptides, which are generally less than 20 amino acids long, play important roles during the life cycle [14]. Recently, it was reported that secreted oligopeptides mediate root-to-shoot signaling, and some long-distance signaling peptides have been detected in xylem exudates [15,16]. In *Lotus japonicus*, CLE-Root Signal 2 functions in the long-distance regulation of nodule formation [15,17]. In Arabidopsis, CEPs mediate root-to-shoot nitrogen deficiency signaling [16], and CLE25 is involved in the systemic drought response [18]. Considering that various internal or external factors affect plant growth and development at the whole-plant level, there are likely additional signaling molecules that are translocated from roots to shoots. We previously developed a gel-free purification system based on a combination of *o*-chlorophenol extraction and high-performance liquid chromatography (HPLC) fractionation [8]. This method is appropriate for the identification of peptide fragments, and seven xylem-associated peptides (XAPs) were identified in soybean (*Glycine max*) xylem exudates. Although these XAPs include CLE and CEP family oligopeptides, the conservation of other possible functional oligopeptides in xylem exudates across plants and the overall features of the identified peptide fragments have been poorly explored.

It is easy to collect xylem exudates from tomato, and a protein database is available for this plant. Furthermore, tomato belongs to the Solanaceae family in the asterid group and is evolutionarily distant from soybean and Arabidopsis, which belong to the rosid group. In this study, by taking advantage of these features, we conducted a comprehensive analysis of small proteins and peptide fragments in tomato xylem exudates using the gel-free purification technique. We first investigated the overall features of peptide fragments in the xylem exudates. Second, we searched for putative functional oligopeptides and analyzed their conservation based on the findings in soybean and Arabidopsis.

## 2. Materials and Methods

### 2.1. Biological Materials and Xylem Exudates Harvesting

To collect xylem sap, *S. lycopersicum* cv. Ponderosa was grown in an open glasshouse with a temperature of approximately 28 °C during the day and 15 °C at night under natural sunlight conditions. The first internodes from the hypocotyls of the plants were cut off at 45 days after germination (DAG), and the exudates were left to drip for 20 min. Then, the cut surface on the root side was washed with distilled water and wiped with paper. The rootstock was attached to a silicon tube, and the gap between the stem and tube was sealed with clay. The xylem exudates were sampled from the stems by root pressure exudation for 5 h after cutting. The exudates were sampled at approximately 30 min intervals from 14 tomato plants. For each sampling, exudates were collected into 15 mL centrifuge tubes on ice and immediately stored at −30 °C.

### 2.2. Protein and Peptide Purification Using a Gel-Free System

The exudates were concentrated to approximately one-tenth of their original volume. The concentrated exudates were partially purified via *o*-chlorophenol extraction and acetone precipitation as described in Ohyama et al. (2009) [19]. The sample was loaded onto a Superdex Peptide 10/300 GL gel filtration column (10 mm × 300 mm; GE Healthcare, Chicago, IL, USA) and eluted with 100 mM ammonium acetate at a flow rate of 0.5 mL/min. Ten fractions were collected from 16 to 36 min. Each small-protein-containing fraction (16 to 24 min) was digested with trypsin, the peptide fragment-containing fractions (24 to 36 min) were lyophilized, and each pellet was dissolved in water. Then, each fraction was loaded onto an Asahipak GS-320 column (7.5 mm × 300 mm; Shodex, Tokyo, Japan) and eluted with 25 mM ammonium acetate at a flow rate of 0.5 mL/min. Ten fractions were collected from 12 to 32 min. Then, the fractions were lyophilized.

### 2.3. Data-Dependent MS/MS Analysis

Nano-LC–MS analysis was performed using an EASY-nLC 1000 (Thermo Fisher Scientific, Waltham, MA, USA) connected to an Orbitrap Elite mass spectrometer (Thermo Fisher Scientific). Small-protein-containing and peptide fragment-containing fractions were loaded onto a trapping column (75 µm i.d. × 20 mm, Acclaim PepMap 100 C18 LC; Thermo Fisher Scientific). Peptides were subsequently eluted from the trapping column and separated on a nanocapillary column (75 µm i.d. × 125 mm, NTCC-360; Nikkyo Technos, Tokyo, Japan) with a 30-min 5–35% acetonitrile (containing 0.1% formic acid) gradient, followed by a 2 min 35–80% gradient, with a final 8 min isocratic step at 80% acetonitrile and a flow rate of 300 nl/min. The nano-HPLC eluate was introduced into a mass spectrometer via an electrospray ionization (ESI) interface at a spray voltage of 2.0 kV. The mass spectrometer was operated in positive ion mode with a capillary temperature of 360 °C. Mass spectra were obtained by scanning from *m/z* 200 to *m/z* 2000. Nano-LC–MS/MS analysis was performed by selecting the indicated molecular ion as the precursor ion at 30% normalized collision energy using the higher energy collision dissociation mode.

The acquired MS/MS spectra were analyzed with MASCOT version 2.8.0.1 using the tomato protein database (ITAG4.0) downloaded from https://phytozome-next.jgi.doe.gov/info/Slycopersicum_ITAG4_0 (accessed on 22 March 2022) [20]. The search was performed using a precursor ion mass tolerance of 10 ppm and a fragment ion mass tolerance of 0.1 Da. For the identification of small proteins, database searches were limited to fully tryptic peptides with two missed cleavages allowed, and carbamidomethyl cysteine; hydroxylated proline; and sulfated tyrosine, serine, and threonine were set as variable modifications (FDR ≤ 0.01). The parent proteins to which more than two unique peptide fragments were matched are listed in Table S1. For the peptidomics analysis, no enzyme was specified, and hydroxylated proline; and sulfated tyrosine, serine, and threonine were set as variable modifications (FDR ≤ 0.01). Datasets of MS/MS spectra have been deposited in the Japan Proteome Standards Repository/Database (jPOST; https://repository.jpostdb.org (accessed on 22 March 2022)) under accession number JPST001538.

### 2.4. Exploration of the Tomato CLE-like Sequence

The tomato genome sequences (ITAG4.0) were scanned for putative CLE homologs by TBLASTN search on the Phytozome v13 platform using the amino acid sequence of Arabidopsis and known tomato CLE precursor polypeptides as queries [21,22,23,24]. The TBLASTN search was repeated using newly identified putative CLE sequences as queries until no additional candidates were identified.

### 2.5. Quantitative PCR Analyses

Total RNA was extracted from the shoot apices, leaves, third internodes, first internodes, and roots of plants at 18 DAG using an ISOGENII (Nippon Gene, Tokyo, Japan), and cDNA was synthesized using ReverTra Ace (Toyobo, Osaka, Japan) following the manufacturer’s protocol. qPCR analysis was performed using Thunderbird Next SYBR qPCR Mix (Toyobo), and the PCR cycling conditions were as follows: a denaturation step at 95 °C for 10 min followed by 45 cycles of 95 °C for 15 s and 60 °C for 1 min. qPCR was performed using the CFX Connect Real-Time PCR system (BioRad, Hercules, CA, USA). The *SlELF1B*-like gene (Solyc01g098000.3.1) was used as a reference gene.

### 2.6. Statistical Analyses

Statistical analyses were performed with R (http://www.R-project.org/ (accessed on 22 March 2022)) and RStudio software (http://www.rstudio.com/ (accessed on 22 March 2022)). Statistical differences in the mRNA levels of genes among organs or tissues were evaluated by one-way ANOVA followed by Tukey’s test.

## 3. Results

### 3.1. Characterization of Small Protein- or Peptide Fragment-Containing Fractions of Tomato Xylem Exudates

**Figure 1:**
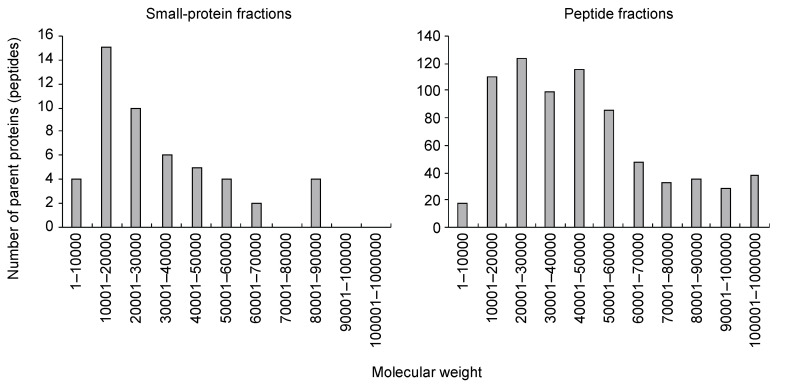
Frequency distribution of the molecular weights of the parent proteins.

We collected 120 mL of xylem exudate from the cut surface of the first internodes from the tomato plant hypocotyls at 45 DAG for five hours. The exudates were purified using the gel-free method that we developed previously [8]. Using gel filtration chromatography, we separated small-protein-containing fractions (small-protein fractions) and oligopeptide-containing fractions (peptide fractions). The small-protein fractions were treated with trypsin, and the peptide fractions were further purified with multimode chromatography. Then, data-dependent MS/MS (ddMS/MS) followed by a MASCOT search was performed using the tomato protein database (ITAG4.0) [20]. In the small-protein fractions, trypsin-digested peptide fragments were assigned to 50 proteins (or peptides) (FDR ≤ 0.01) (Table S1). Some of these proteins were annotated as LTPs or peroxidases, which have been commonly identified in xylem exudates from other plants. A high proportion of the proteins had molecular weights (MWs) of 10,000 to 20,000 (Figure 1), as determined by gel filtration chromatography. On the other hand, in the peptide fractions, peptide fragments were assigned to 729 proteins (or peptides) (FDR ≤ 0.01) (Table S2). The MWs of the parent proteins varied (Figure 1), suggesting that fragmented proteins—but not full-length proteins—were present in the fractions.

To characterize the assigned proteins, we searched for putative secretion signals using SignalP 3.0 (https://services.healthtech.dtu.dk/service.php?SignalP-3.0 (accessed on 22 March 2022)) [25]. As a result, 66.0% and 39.4% of the parent proteins were predicted to be secreted proteins in the small-protein and peptide fractions, respectively (Table 1). In contrast, 16.3% of the proteins in the tomato database (ITAG4.0) were predicted to be secreted proteins. These results indicate that secreted proteins were enriched in the xylem exudates.

We further conducted gene ontology (GO) enrichment analysis of the parent proteins (peptides) using Shiny GO v0.75 (http://bioinformatics.sdstate.edu/go/ (accessed on 22 March 2022)) (FDR ≤ 0.01) [26], and the GO terms were divided into biological processes, cellular components, and molecular functions. In the small-protein fractions, GO-enriched terms were related to defense against biotic stresses and metabolism of reactive oxygen species (Figure S1). In contrast, in the peptide fractions, the enriched GO categories included carbohydrate metabolism and proteolysis (Figure S2).

### 3.2. Identification of Tomato Xylem Exudate-Associated Oligopeptides

It has been reported that bioactive oligopeptides mediate cell-to-cell or organ-to-organ signaling during plant life [27,28,29,30] and that such functional oligopeptides have several features in precursor proteins (peptides) as follows [14]: (1) amino acid sequences of functional peptide-encoding regions are well conserved across plant species; (2) precursor proteins (peptides) possess secretion signals; and (3) the precursor proteins (peptides) are usually less than 200 amino acids in length. Based on these molecular features, we searched for possible functional oligopeptides in tomato xylem exudates. As a result, we found six endogenous oligopeptides (Table 2, Figure S3). These peptides were conserved in soybean, Arabidopsis, poplar (*Populus trichocarpa*), and rice (*Oryza sativa*) (Figure S4). Solyc03g098750.3.1 and Solyc06g052020.2.1 are homologs of GmXAP1 and GmXAP5, respectively, which were previously identified in soybean xylem exudates [8]. In addition, Solyc01g109895.1.1 (SlCIF1) is a homolog of GmXAP3 and Arabidopsis CIF peptides [31,32]. Solyc02g090600.2.1 (SlCEP1) is a CEP, although the obtained amino acid sequence did not completely correspond to the CEP domain. Solyc07g150120.1.1 and Solyc03g115950.3.1 are homologous peptides, but their homologs have not been detected in xylem exudates of other plants.

Meanwhile, we could not identify CLE peptides in the tomato xylem exudates. Some of these peptides are known to function in root-to-shoot signaling [15,18], and a CLE peptide was identified in soybean xylem exudates [8]. Although 15 CLEs are included in the tomato protein database [24], considering that there are 32 *CLE* genes in Arabidopsis [23], more *CLE* genes are likely present in the tomato genome. To further search for *CLE*-like sequences, we performed TBLASTN searches against the tomato genome database on the Phytozome v13 platform using full-length Arabidopsis and known tomato CLE precursor proteins as queries [21,22]. We found 22 *CLE*-like sequences, and those putative amino acid sequences are shown in Table S3. Then, we added these amino acid sequences to the protein database and reanalyzed them with MASCOT software. As a result, we identified the mature SlCLE20 peptide from tomato xylem exudates (Table 2). SlCLE20 showed high homology to GmXAP4/GmCLE32 and Arabidopsis CLE2 peptides (Figure S4). In those peptides, the seventh hydroxyproline was modified with three residues of arabinose [8,19], and we confirmed that the seventh hydroxyproline in SlCLE20 was also arabinosylated (Figure S3).

### 3.3. Expression Analysis of the Oligopeptide-Encoding Genes

**Figure 2:**
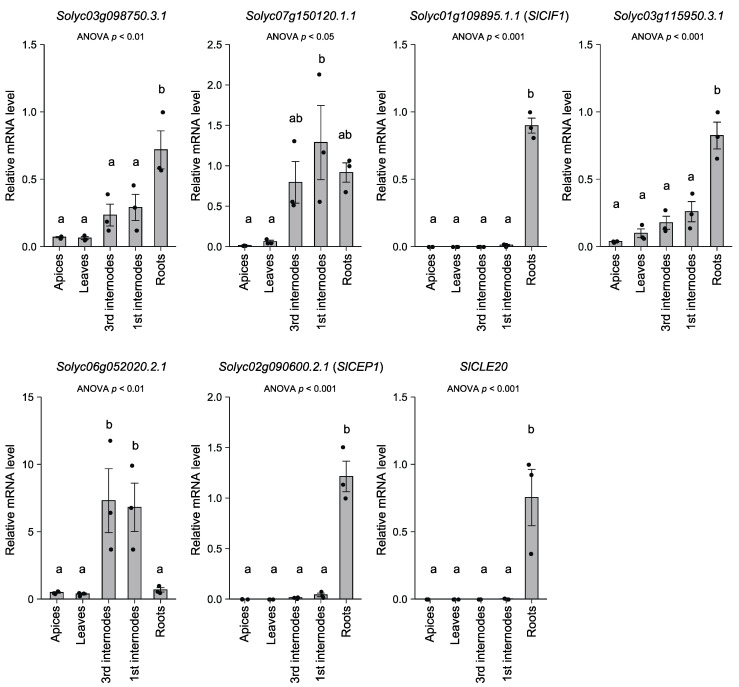
Relative expression of oligopeptide-encoding genes in various tissues.

We further explored the mRNA level of the seven oligopeptide-encoding genes in apices, leaves, and first (mature) and third (young) internodes of the hypocotyls and roots of tomato plants (19 DAG) (Figure 2). Interestingly, the expression of *Solyc01g109895.1.1* (*SlCIF1*), *Solyc02g090600.2.1* (*SlCEP1*), and *SlCLE20* was detected exclusively in roots. The mRNA levels of *Solyc03g098750.3.1* and *Solyc03g115950.3.1* were significantly higher in roots. In contrast, the mRNA levels of *Solyc07g150120.1* and *Solyc06g0520202.1* were higher in the first and third internodes.

The relative mRNA levels in each organ or tissue are based on those in the roots. Dots represent individual measurements. Statistical differences were evaluated by one-way ANOVA followed by Tukey’s test. Difference of alphabet letters indicates statistically significant difference (*p* < 0.05). Each result is the mean ± SE of measurements obtained from two individual samples in three independent experiments. The gene-specific primers used for qPCR are listed in Table S4.

## 4. Discussion

In this study, we conducted a comprehensive analysis of small proteins and peptides in tomato xylem exudates using gel-free methods. As a result, 50 and 729 proteins (or peptides) were assigned in the small-protein and peptide fractions, respectively. In the small-protein fractions, the MWs of more than half of the parent proteins were ≤30,000 (Figure 1). This suggests that the small proteins were full-length proteins, although the secretion signal was likely truncated, and were fractionated by gel filtration chromatography. As reported in other plants [1,3,4,5,6,7], LTPs and peroxidases were identified in tomato xylem exudates. GO analysis showed that these fractions were enriched with terms related to defense against biotic stresses and metabolism of reactive oxygen species (Figure S1). Reactive oxygen species are toxic compounds that can affect pathogens, and some peroxidases are suggested to be involved in cell wall modification [33]. The xylem is a target of most vascular pathogens [34], so it seems that plants express defense proteins against unexpected pathogen invasions.

On the other hand, in the peptide-containing fractions, the obtained peptide fragments were assigned to proteins of various sizes (Figure 1). These fractions were not treated with trypsin, so the parent proteins were likely already fragmented before sampling. Many of the parent proteins were annotated as proteins that function as full-length proteins, such as receptor-like protein kinases and subtilases. These results suggest that the peptide fragments derived from those proteins may drift in xylem exudates as degradation products of proteins rather than as signaling molecules.

Among the assigned proteins, 66.0% and 39.4% were predicted to be secreted proteins in the small-protein and peptide fractions, respectively (Table 1). Considering that only 16.3% of the proteins in the tomato database were predicted to be secreted proteins, secreted proteins were highly enriched in xylem exudates. Considering that the xylem conduit consists of empty dead cells and is a kind of apoplast, it seems that our result at least partly reflects the protein composition of the xylem sap.

However, the remaining parent proteins were not predicted to be secreted proteins. Although some of them might actually be secreted proteins, considering that some proteins were annotated as intracellular proteins, such as actins and ribosomal proteins, those proteins might have been derived from dead or injured cells in roots or cut stems. Meanwhile, in animals, extracellular vesicles (EVs) are secreted from cells and mediate not only local communication but also long-distance communication through blood vessels [35]. EVs contain proteins and nucleic acids that are usually localized to subcellular regions or cell membranes. Furthermore, in plants, EV-like structures have been observed in xylem and phloem vessels [36]. Therefore, there is a possibility that some of the nonsecreted proteins might have been derived from EVs. To test this hypothesis, purification of EVs and proteome analysis should be conducted.

Recently, it has been revealed that small, secreted peptides mediate cell-to-cell and organ-to-organ communications in plants [27,28,29,30]. Despite their biological importance, peptide-encoding short open reading frames (ORFs) are often excluded from protein databases to avoid a high rate of false-positive ORF predictions because short putative ORFs can be expected to be present in long noncoding sequences [37]. To date, 15 *CLE* genes have been reported in tomato [24], and we newly identified 22 *CLE*-like sequences in the tomato genome (Table S3). By adding the amino acid sequences of these putative CLE precursor proteins to the reference protein database, we identified the mature SlCLE20 peptide in xylem exudates. We found that the *SlCLE20* gene was mainly expressed in roots (Figure 2), although the expression of other *CLE*-like sequences remains unexplored. Hanada et al. (2007) developed a computational method to find possible short ORFs [38], and some of the identified short ORFs led to visible phenotypes when they were overexpressed [39]. Improvement of the protein database is important for more comprehensive identification of possible functional peptides, and such a data mining method may contribute to related analysis.

We identified seven endogenous oligopeptides in tomato xylem exudates (Table 2). Interestingly, five of them were homologs of soybean xylem exudate-associated peptides [8]. Tomato is a member of the asterid group and is evolutionarily distant from soybean and Arabidopsis, which belong to the rosid group. Our results suggest that oligopeptides are conserved in xylem exudates across plant species and that they play general roles rather than have species-specific functions. However, we must note that *Solyc07g150120.1.1* and *Solyc06g052020.2.1* were highly expressed in the first and third internodes (Figure 2). This suggests that these oligopeptides were derived from the cut surface of internodes, although we cannot eliminate the possibility that they were translocated from the lower part of the stem to upper organs or tissues in shoots.

In contrast, the mRNA levels of the other five endogenous peptide-encoding genes were higher in roots (Figure 2). In particular, *Solyc01g109895.1.1* (*SlCIF1*), *Solyc02g090600.2.1* (*SlCEP1*), and *SlCLE20* were exclusively expressed in roots, so it appears that these oligopeptides were derived from roots. Among the CLE peptides, the SlCLE20 peptide showed high homology with GmXAP4/GmCLE32 and Arabidopsis CLE1 to 7. It has been reported that the *CLE1* to *7* genes respond to various stimuli, such as nitrogen, salt, low temperature, and sulfate [40,41,42]. On the other hand, some CEPs are known to respond to nitrogen deficiency and function in root-to-shoot signaling [16]. In addition, *CEP* genes respond to various abiotic stress conditions [43]. Therefore, SlCLE20 and Solyc02g0906002.1 (SlCEP1) could be involved in the systemic response to external or internal stimuli.

CIF1 and CIF2 are expressed in the root stele and are required for Casparian strip formation in roots [31,32]. A functional model was proposed: when these peptides diffuse into the endodermal region, they are recognized by GASSHO1(GSO1)/SCHENGEN3 and GSO2 receptor-like kinases and induce Casparian strip formation. Furthermore, it is mentioned that CIF peptides function not only in the de novo formation of the Casparian strip, but also in its maintenance. In our ddMS/MS analysis, many SlCIF1 (Solyc01g109895.1.1) peptides were detected. This suggests that xylem exudates contain many CIF peptides, and it seems that CIF peptides are continually supplied to the xylem stream to survey breakage of the Casparian strip. To clarify the biological roles of these tomato xylem exudate-associated oligopeptides, detailed analyses should be conducted, and the Micro-Tom model system would be helpful for the analyses. Tomato is an important crop plant, and findings on long-distance mobile peptides might contribute to the improvement of its yield at the whole-plant level.

## 5. Conclusions

In this study, we identified various small proteins and peptide fragments in tomato xylem exudates and found that putative secreted proteins (peptides) were enriched in the exudates. In the peptide fraction, although a large portion of the peptides was likely derived from protein degradation, we found seven endogenous oligopeptides that showed common features of functional oligopeptides. These five oligopeptides were homologs of soybean xylem exudate-associated oligopeptides, suggesting that oligopeptides in xylem exudates are conserved across plant species. Considering the characteristics of the identified oligopeptides, our results suggest that they function in not only root-to-shoot signaling, but also the maintenance of plant apoplastic structures.

## Figures and Tables

**Table 1:** Number and ratio of putative secreted proteins.

	Secreted Proteins	Nonsecreted Proteins
Small-protein fractions	33 (66.0%)	17 (34.0%)
Peptide fractions	287 (39.4%)	442 (60.6%)
Tomato protein database (ITAG 4.0)	5563 (16.3%)	28,511 (83.7%)

**Table 2:** Oligopeptides identified in tomato xylem exudates.

Accession Number in ITAG4.0	MW of Precursor Protein	Amino Acid Sequence	Note
Solyc03g098750.3.1	11,668	DyAGTGANNHHDPKPpGSQW ^†^	Homolog of GmXAP1
Solyc07g150120.1.1	9252	DyPSSGANNRHTpSHP ^†^	---
Solyc01g109895.1.1 (SlCIF1)	13,357	DyGRYDPTpALSKPPFKLIPN ^†^	Homolog of GmXAP3
Solyc03g115950.3.1	10,409	DyPGSGANNRHTp ^†^	---
Solyc06g052020.2.1	8649	DYDNAGpNTKHD	Homolog of GmXAP5
Solyc02g090600.2.1 (SlCEP1)	18,882	YApKQTGNSpGIGHSS	Homolog of GmXAP6
(SlCLE20)	8402	RVSpGGp*DPHHH	Homolog of GmXAP4

^†^ The identified amino acid sequence varied, so a representative sequence is shown. Modifications: p (hydroxylation), p* (hydroxylation and arabinosylation), y (sulfation).

## Data Availability

Datasets of MS/MS spectra are openly available in the Japan Proteome Standards Re-pository/Database (jPOST; https://repository.jpostdb.org, accessed on 22 March 2022) under accession number JPST001538.

## Supplementary Materials

The following supporting information can be downloaded at: https://www.mdpi.com/article/10.3390/life12040592/s1. Table S1. List of proteins whose peptide fragments were identified in the small-protein-containing fractions; Table S2. List of the proteins whose peptide fragments were identified in peptide-containing fractions; Table S3. Newly predicted tomato CLE-like sequences; Table S4. List of PCR primers; Figure S1. GO analysis of the parent proteins in small-protein fractions; Figure S2. GO analysis of the parent proteins in peptide fractions; Figure S3. MS/MS spectra of the seven tomato xylem exudate-associated oligopeptides; Figure S4. Amino acid sequence alignment of the precursor polypeptides of tomato xylem exudate-associated peptides and their homologs in various plants.

## References

[1] Biles C.L., Abeles F.B. (1991). Xylem Sap Proteins. Plant Physiol..

[2] Iwai H., Usui M., Hoshino H., Kamada H., Matsunaga T., Kakegawa K., Ishii T., Satoh S. (2003). Analysis of Sugars in Squash Xylem Sap. Plant Cell Physiol..

[3] Buhtz A., Kolasa A., Arlt K., Walz C., Kehr J. (2004). Xylem Sap Protein Composition Is Conserved among Different Plant Species. Planta.

[4] Kehr J., Buhtz A., Giavalisco P. (2005). Analysis of Xylem Sap Proteins from Brassica Napus. BMC Plant Biol..

[5] Alvarez S., Goodger J.Q.D., Marsh E.L., Chen S., Asirvatham V.S., Schachtman D.P. (2006). Characterization of the Maize Xylem Sap Proteome. J. Proteome Res..

[6] Djordjevic M.A., Oakes M., Li D.X., Hwang C.H., Hocart C.H., Gresshoff P.M. (2007). The Glycine Max Xylem Sap and Apoplast Proteome. J. Proteome Res..

[7] Ligat L., Lauber E., Albenne C., San Clemente H., Valot B., Zivy M., Pont-Lezica R., Arlat M., Jamet E. (2011). Analysis of the Xylem Sap Proteome of Brassica Oleracea Reveals a High Content in Secreted Proteins. Proteomics.

[8] Okamoto S., Suzuki T., Kawaguchi M., Higashiyama T., Matsubayashi Y. (2015). A Comprehensive Strategy for Identifying Long-Distance Mobile Peptides in Xylem Sap. Plant J..

[9] Luo J.-S., Zhang Z. (2019). Proteomic Changes in the Xylem Sap of Brassica Napus under Cadmium Stress and Functional Validation. BMC Plant Biol..

[10] Young S.A., Guo A., Guikema J.A., White F.F., Leach J.E. (1995). Rice Cationic Peroxidase Accumulates in Xylem Vessels during Incompatible Interactions with Xanthomonas Oryzae Pv Oryzae. Plant Physiol..

[11] Marjamaa K., Kukkola E.M., Fagerstedt K.V. (2009). The Role of Xylem Class III Peroxidases in Lignification. J. Exp. Bot..

[12] Maldonado A.M., Doerner P., Dixon R.A., Lamb C.J., Cameron R.K. (2002). A Putative Lipid Transfer Protein Involved in Systemic Resistance Signalling in Arabidopsis. Nature.

[13] Chanda B., Xia Y., Mandal M.K., Yu K., Sekine K., Gao Q., Selote D., Hu Y., Stromberg A., Navarre D. (2011). Glycerol-3-Phosphate Is a Critical Mobile Inducer of Systemic Immunity in Plants. Nat. Genet..

[14] Matsubayashi Y. (2011). Post-Translational Modifications in Secreted Peptide Hormones in Plants. Plant Cell Physiol..

[15] Okamoto S., Shinohara H., Mori T., Matsubayashi Y., Kawaguchi M. (2013). Root-Derived CLE Glycopeptides Control Nodulation by Direct Binding to HAR1 Receptor Kinase. Nat. Commun..

[16] Tabata R., Sumida K., Yoshii T., Ohyama K., Shinohara H., Matsubayashi Y. (2014). Perception of Root-Derived Peptides by Shoot LRR-RKs Mediates Systemic N-Demand Signaling. Science.

[17] Okamoto S., Ohnishi E., Sato S., Takahashi H., Nakazono M., Tabata S., Kawaguchi M. (2009). Nod Factor/Nitrate-Induced CLE Genes That Drive HAR1-Mediated Systemic Regulation of Nodulation. Plant Cell Physiol..

[18] Takahashi F., Suzuki T., Osakabe Y., Betsuyaku S., Kondo Y., Dohmae N., Fukuda H., Yamaguchi-Shinozaki K., Shinozaki K. (2018). A Small Peptide Modulates Stomatal Control via Abscisic Acid in Long-Distance Signalling. Nature.

[19] Ohyama K., Shinohara H., Ogawa-Ohnishi M., Matsubayashi Y. (2009). A Glycopeptide Regulating Stem Cell Fate in Arabidopsis Thaliana. Nat. Chem. Biol..

[20] Hosmani P.S., Flores-Gonzalez M., van de Geest H., Maumus F., Bakker L.V., Schijlen E., van Haarst J., Cordewener J., Sanchez-Perez G., Peters S. (2019). An Improved de Novo Assembly and Annotation of the Tomato Reference Genome Using Single-Molecule Sequencing, Hi-C Proximity Ligation and Optical Maps. bioRxiv.

[21] Altschul S.F., Madden T.L., Schäffer A.A., Zhang J., Zhang Z., Miller W., Lipman D.J. (1997). Gapped BLAST and PSI-BLAST: A New Generation of Protein Database Search Programs. Nucleic Acids Res..

[22] Goodstein D.M., Shu S., Howson R., Neupane R., Hayes R.D., Fazo J., Mitros T., Dirks W., Hellsten U., Putnam N. (2012). Phytozome: A Comparative Platform for Green Plant Genomics. Nucleic Acids Res..

[23] Cock J.M., McCormick S. (2001). A Large Family of Genes That Share Homology WithCLAVATA3. Plant Physiol..

[24] Zhang Y., Yang S., Song Y., Wang J. (2014). Genome-Wide Characterization, Expression and Functional Analysis of CLV3/ESR Gene Family in Tomato. BMC Genom..

[25] Dyrløv Bendtsen J., Nielsen H., von Heijne G., Brunak S. (2004). Improved Prediction of Signal Peptides: SignalP 3.0. J. Mol. Biol..

[26] Ge S.X., Jung D., Yao R. (2020). ShinyGO: A Graphical Gene-Set Enrichment Tool for Animals and Plants. Bioinformatics.

[27] Tavormina P., De Coninck B., Nikonorova N., De Smet I., Cammue B.P.A. (2015). The Plant Peptidome: An Expanding Repertoire of Structural Features and Biological Functions. Plant Cell.

[28] Notaguchi M., Okamoto S. (2015). Dynamics of Long-Distance Signaling via Plant Vascular Tissues. Front. Plant Sci..

[29] Okamoto S., Tabata R., Matsubayashi Y. (2016). Long-Distance Peptide Signaling Essential for Nutrient Homeostasis in Plants. Curr. Opin. Plant Biol..

[30] Takahashi F., Hanada K., Kondo T., Shinozaki K. (2019). Hormone-like Peptides and Small Coding Genes in Plant Stress Signaling and Development. Curr. Opin. Plant Biol..

[31] Nakayama T., Shinohara H., Tanaka M., Baba K., Ogawa-Ohnishi M., Matsubayashi Y. (2017). A Peptide Hormone Required for Casparian Strip Diffusion Barrier Formation in Arabidopsis Roots. Science.

[32] Doblas Verónica G., Smakowska-Luzan E., Fujita S., Alassimone J., Barberon M., Madalinski M., Belkhadir Y., Geldner N. (2017). Root Diffusion Barrier Control by a Vasculature-Derived Peptide Binding to the SGN3 Receptor. Science.

[33] Passardi F., Penel C., Dunand C. (2004). Performing the Paradoxical: How Plant Peroxidases Modify the Cell Wall. Trends Plant Sci..

[34] Yadeta K., Thomma B. (2013). The Xylem as Battleground for Plant Hosts and Vascular Wilt Pathogens. Front. Plant Sci..

[35] Yáñez-Mó M., Siljander P.R.-M., Andreu Z., Bedina Zavec A., Borràs F.E., Buzas E.I., Buzas K., Casal E., Cappello F., Carvalho J. (2015). Biological Properties of Extracellular Vesicles and Their Physiological Functions. Null.

[36] Chukhchin D.G., Bolotova K., Sinelnikov I., Churilov D., Novozhilov E. (2019). Exosomes in the Phloem and Xylem of Woody Plants. Planta.

[37] Dinger M.E., Pang K.C., Mercer T.R., Mattick J.S. (2008). Differentiating Protein-Coding and Noncoding RNA: Challenges and Ambiguities. PLoS Comput. Biol..

[38] Hanada K., Zhang X., Borevitz J.O., Li W.-H., Shiu S.-H. (2007). A Large Number of Novel Coding Small Open Reading Frames in the Intergenic Regions of the Arabidopsis Thaliana Genome Are Transcribed and/or under Purifying Selection. Genome Res..

[39] Hanada K., Higuchi-Takeuchi M., Okamoto M., Yoshizumi T., Shimizu M., Nakaminami K., Nishi R., Ohashi C., Iida K., Tanaka M. (2013). Small Open Reading Frames Associated with Morphogenesis Are Hidden in Plant Genomes. Proc. Natl. Acad. Sci. USA.

[40] Araya T., Miyamoto M., Wibowo J., Suzuki A., Kojima S., Tsuchiya Y.N., Sawa S., Fukuda H., von Wirén N., Takahashi H. (2014). CLE-CLAVATA1 Peptide-Receptor Signaling Module Regulates the Expansion of Plant Root Systems in a Nitrogen-Dependent Manner. Proc. Natl. Acad. Sci. USA.

[41] Dong W., Wang Y., Takahashi H. (2019). CLE-CLAVATA1 Signaling Pathway Modulates Lateral Root Development under Sulfur Deficiency. Plants.

[42] Ma D., Endo S., Betsuyaku S., Shimotohno A., Fukuda H. (2020). CLE2 Regulates Light-Dependent Carbohydrate Metabolism in Arabidopsis Shoots. Plant Mol. Biol..

[43] Aggarwal S., Kumar A., Jain M., Sudan J., Singh K., Kumari S., Mustafiz A. (2020). C-Terminally Encoded Peptides (CEPs) Are Potential Mediators of Abiotic Stress Response in Plants. Physiol. Mol. Biol. Plants.

