# Supplementary material for: Characterization of Oligopeptides in Solanum lycopersicum Xylem Exudates
Source: Life (Basel). 2022 Apr 16;12(4):592. doi: 10.3390/life12040592 (PMC9028419; doi:10.3390/life12040592)
Supplement: Supplementary file 1 [file life-12-00592-s001.zip › Figures_Supplement_R.pdf]

Figure S1

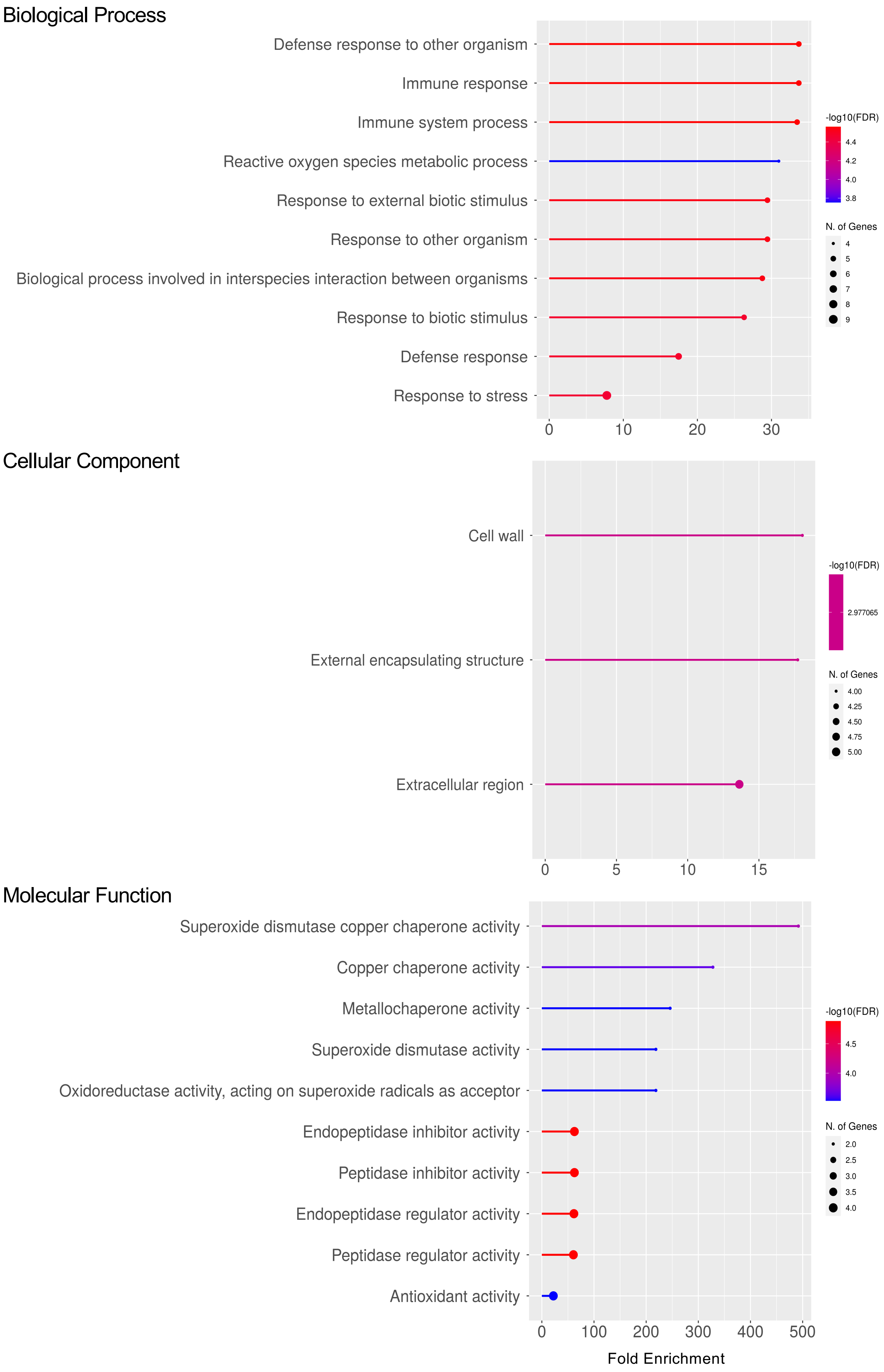

Figure S1. GO analysis of the parent proteins in small-ptotein fractions. Ten of the top pathways are shown in each category.

Figure S2

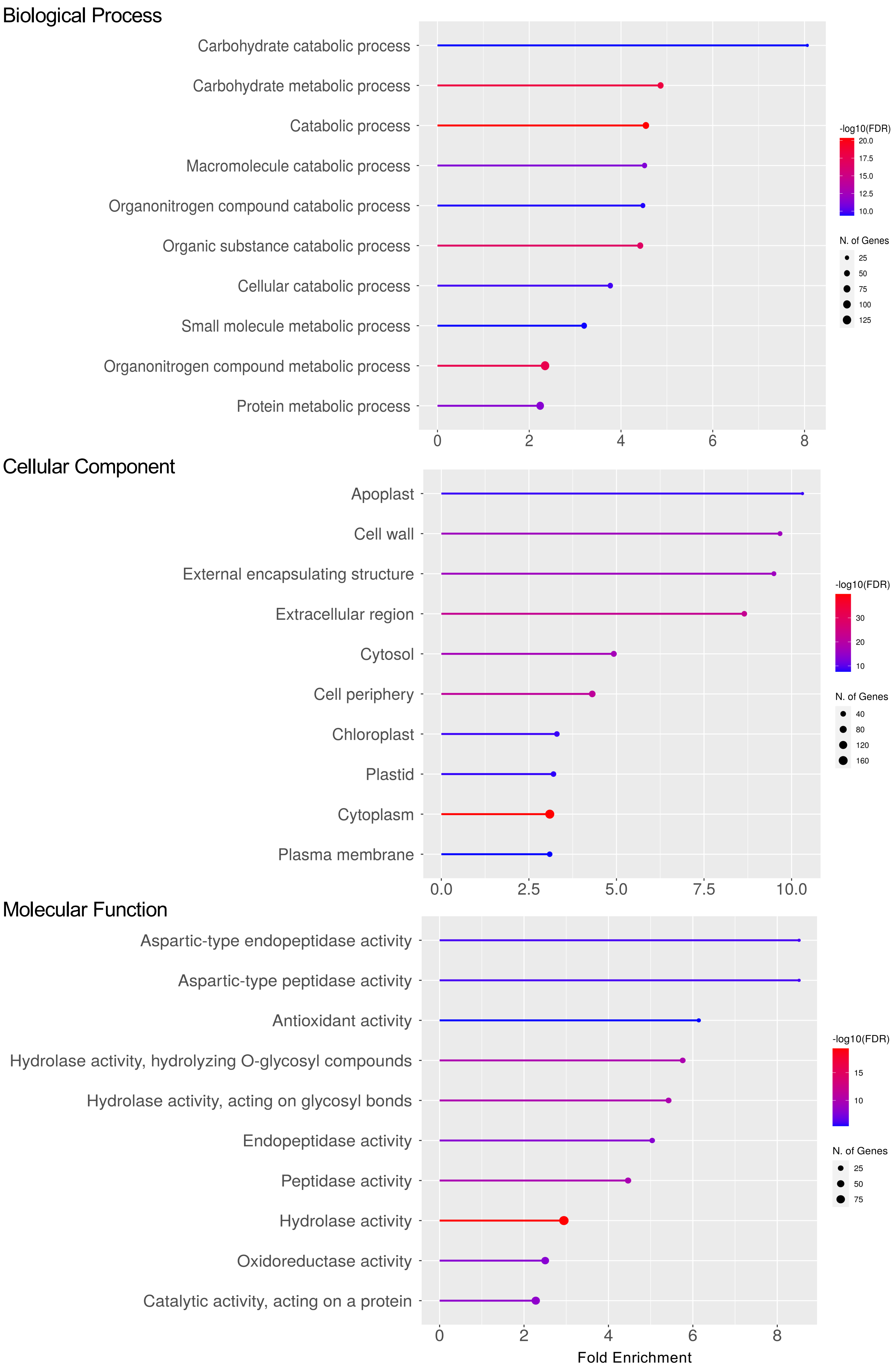

Figure S2. GO analysis of the parent proteins in peptide fractions. Ten of the top pathways are shown in each category.

Figure S3

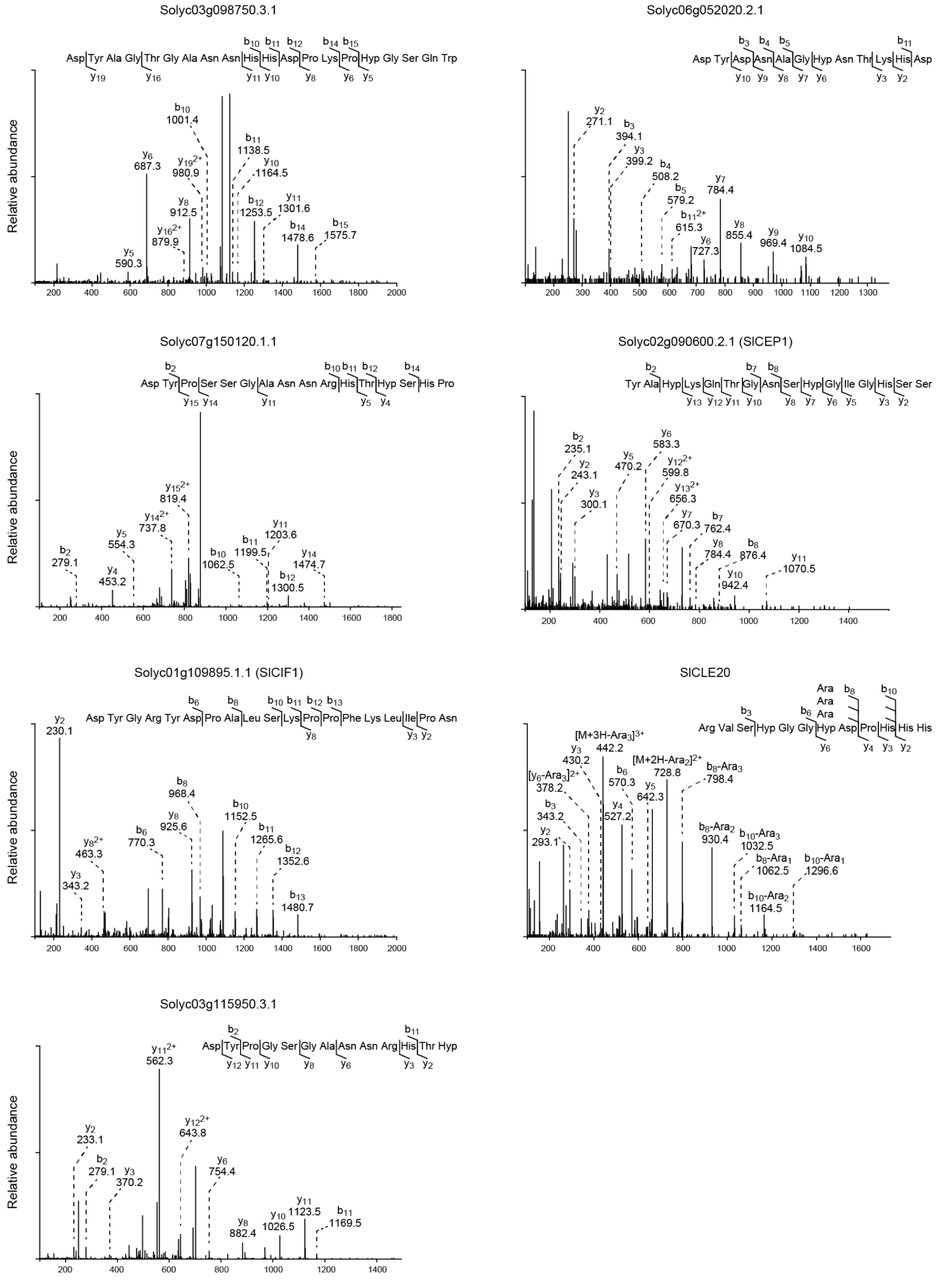

Figure S3. MS/MS spectra of the seven tomato xylem exudate-associated oligopeptides.

Figure S4

```
Solyc03g098750.3.1            -----MEKKVSFLSLLLLIFVGFSLLISTIAVPSSR--SIKTN-----------
Glyma.19G117800.1(GmXAP1)     -----MLRKLMRLLLLLMFFLCFSYVLSVSAIPATRTQNLKGEEEEFSALPSLT
Potri.013G066600.1            ---------MAGSLRLLALLLALSFVLSSVAVPATR--SLKSNDEIPSEVQDLL
AT3G47510.1                   -------MANKALIHLLVIFFLALSHIFFFPSHASRLGSLMEKPKIVR------
LOC_Os01g59970.1              MKMEQVSQRKAPLLVLVILCGLLLLLPLVSSVPLSRSVSLRNHQASVS------
                                             *: :           .   :*  .:  .

Solyc03g098750.3.1            -TENQVSHDKVVLMDELE--KKRELLVERDDYAGTGANNHHDPKPPGSQWSNLN
Glyma.19G117800.1(GmXAP1)     RLDHVVGNGEVVLVDMNEGFMERRVDLETQDYEGTGANKDHDPKSPGGA-----
Potri.013G066600.1            PQDAVISTDGEMLIDAGEGYIEGRMDLESTDYPGTGANNHHDPKTPGKA-----
AT3G47510.1                   PQQDTLMDVKKEVEER--------MVMELNDYPGSGANNRHLPRQRGCVVDC--
LOC_Os01g59970.1              ALEAPVQVVAAAAEERDLDEVAARMVIEVNDYPGSGANNRHDPKSPGRA-----
                                :  :        :         : :*  ** *:***: * *:  *


Solyc03g115950.3.1            ------MAAPFFRLFISFLCFSYTINSFINAVPVTRSKSLVLLDISQEHNVLS
Potri.015G062300.1            ------MAGMLFRLLVSFLLISNLVYWSNISISTRRTGSQVHGHDQVPPILDD
Solyc07g150120.1.1            ---------MATTLFLAFLCFSQVIYLN--TIPITRCSNLVYKAQQHENTRTM
LOC_Os01g59970.1              MKMEQVSQRKAPLLVLVILCGLLLLLPLVSSVPLSRSVSLRNHQASVSALEAP
Glyma.03G007400.1             MRFKSPLQLLIILLVFSFVLSSAALQTTRRLLPNKEKTPTQITSDKFQGVEEL
AT1G74458.1                   ---MARRQLFYHRMFLVLALLYLMISWKPLLITARPEKSTDFETKGQELNRQI
                                           :.. :      :      :.

Solyc03g115950.3.1            ENIILMDKTR---REVLEAEEIIN--DYPGSGANNRHTPRPQLGRGCVEC
Potri.015G062300.1            THLVLQSKKKDHDEHIAHGRKIVELNDYPGSGANNRHTPRPQFGR-CVDC
Solyc07g150120.1.1            ENIEAENMKLG---DVVIRRMDVEVNDYPSSGANNRHTPSHP--------
LOC_Os01g59970.1              VQVVAAAAEER-DLDEVAARMVIEVNDYPGSGANNRHDPKSPGRA-----
Glyma.03G007400.1             KNGEE--MFDMAEEFMVERRIDLESNDYPGTGANNRHDPKTPGGP-----
AT1G74458.1                   SHLGRIENDPERRKGTRRMMTVEDINDYPGSGANNRHTPHCSDC------
                               :                     :  ***.:****** *


Solyc01g109895.1.1(SlCIF1)    -MGGLMLIKKISLFFLLISASLFSTSFAGRQSSFLSNFHQEELS--ATHE
Glyma.13G201100.1(GmXAP3)     -MGTLMLLKKFTLLFLLISGSLLSTSFAGRVRSNSIKNFAKDVN--AIEE
Potri.009G119900.2            --MGLMLLKKISLLFLLISASFLSTSFAGRRSKSVNKLAEEVEVSAATYE
AT2G16385.1(CIF1)             MGMSPLTVKKLGFIFMIVSASALSVSFAGRPSIFVHKKINLREE------
LOC_Os11g21804.1              -MSSRRVNRASVFVILLIVASALSVFTAGGRELVAQETNQKKYSSAALGE
                                      :   :.::::.*  :*.  **       :

Solyc01g109895.1.1 (SlCIF1)    ----------EEQLRHERVLKMNTKDYGRYDPTPALSKPPFKLIPN
Glyma.13G201100.1(GmXAP3)     GTPRKPLNKEEVRTIHERLLRANTKDYGRYDPSPSLSKPPFKLIPN
Potri.009G119900.2            EISSKPSHNNEATTIHERLLKANTKDYGNYKPAPALVRPPFKLIPN
AT2G16385.1(CIF1)             -------MVERSMHEHERLLRMNTKDYGNNSPSPRLERPPFKLIPN
LOC_Os11g21804.1              G-------ATSSGEVHPRNLMVKTNDYGRYDPSPAFSKPRFKIIPN
                                             * * *  :*:***. .*:* : :* **:***


Solyc06g052020.2.1            ---------MRPRRIKALILLLLFLIFFSS--GHVEG-----------VN
AT5G53486.1                   ----------MPLRIEPLSLLFLILIICSSSLAIVEGSIQLDGAKMHESS
Potri.012G017400.2            -------MALTLGMIKTFLLVSLLLLAPFSS-GFVEG------FDEGMN-
Glyma.04G233800.3(GmXAP5)     ----------MALKINTFLLVLLLILIPLSS-GLAEG------FGENMHP
LOC_Os05g46470.1              MPSVSVASSMSKLKFLVTVVLVILLLFDSSSSGQAET----------AYT
                                            :    :: ::::   *  . .*

Solyc06g052020.2.1            RAYKLHKHVIGENPRKLLQLDTVLDYDNAGPNTKHDPRGKKGGSSGNHP
AT5G53486.1                   MLYKSEEVGVKKMGHRKLMFHSTADYDDAGPNPKHDPRRRPGGKG----
Potri.012G017400.2            SYPSLHKDGIQVNTRKLLLVD-ELDYDDAGANRRHDPRGRPGVGGYKNP
Glyma.04G233800.3(GmXAP5)     THGLLYKDGIKMNSRKLLVHDFVLDYDEAGPNPRHTKK--PGKGP----
LOC_Os05g46470.1              NYEVTSKVKERIQKHSRRVLTDVQDYDYGGPNPRHEPRKKPGNGH----
                                    :       :         *** .*.* :*  :   *
```

Continued on the next page

Figure S4 (continued)

```
Solyc02g090600.2.1(SlCEP1)      ------------MQQICLFSFIVAVIACHLVPFAEGRQLKELKKPKLADN---
Glyma.01G185000.1(GmXAP6)       --------MDEFQTMHKYFTIFVALFACHGSLFAHGRQIKPLNQHSSLNTNPI
AT5G66816.1(CEP6)               ----------MKLSVYIILSILFISTVFYEIQFTEARQLRKTDDQ--------
SLOC_Os03g27680.1                MKGKRTFLSSLNKEHIKKFYVLERVVAQFYLFSSQGRPLPDDDGITSEMQIR-
                                                 : .:    . .    :..* :    .

Solyc02g090600.2.1 (SlCEP1)     -----------MSANQILEKQRFTIRDASKVVNGKINYAPKQTGNSPGIGHSS
Glyma.01G185000.1(GmXAP6)       LAPLSRTSIKVIEAPIVPKFKFSDVDSGDSGADHANAFRPTTPGNSPGVGHKK
AT5G66816.1(CEP6)               -----------------------DHDDHHFTVGYTDDFGPTSPGNSPGIGHKM
LOC_Os03g27680.1                ------RYLLSHGNGVVEGAVSPSSEIGGPMVGASGGVRPTNPGHSPGIGHHV
                                                               ..      *. .*:***:**

Solyc02g090600.2.1 (SlCEP1)     SVPKKFNY---TPNQTGNSPGIGHSSSVP------------------KTNYQ
Glyma.01G185000.1(GmXAP6)       FEEDKVMK---VMGALVHSPDV-----------------------------KTS
AT5G66816.1(CEP6)               KENE-------------------------------------------------
LOC_Os03g27680.1                AINGDVDDDDVRPTNPGHSPGIGHHAIVNGADDADDVRPTNPGHSPGIGHAVV

Solyc02g090600.2.1 (SlCEP1)     TKHTNVEKSIDDIKAGHSPGIGHSSQSEKIGPNV----
Glyma.01G185000.1(GmXAP6)       VAEGSFENDFKPTDPGHSPGVGHPRQNKRN--------
AT5G66816.1(CEP6)               ENAGGYKDDFEPTTPGHSPGVGHAVKNNEPNA------
LOC_Os03g27680.1                NSADDDADDVRPTNPGHSPGIGHAFVNKIDGPAGKKKL
                                    .  ...    .*****:**.   .:


SlCLE20                         -MINTMSKFKYLSCIILLLLSSALLEARPIR-----------SIRIAREA
Glyma.13G171400.1(GmXAP4)       ----MAMLTRFLGCICLILILSAGSEARPLNPT---------IVRDVLGT
AT4G18510.1(CLE2)               ----MAKLSFTFCFLLFLLLSSIAAGSRPLEGARVGVK--VRGLSPSIEA
Potri.019G091100.1              -----MANLKLWVCLLLLFLTFSKSETRLLDQPYLGRKNPARMLQELNEK
LOC_Os01g55080.1                MAARTYLAVFAFCAILMLSAVARSASARALRERAPEEAAVVAGRAGVAGM
                                              : ::        :*  :

SlCLE20                         LEEQFEREKAKVG--------------AHEWPQRVSPGGPDPHHHFKNN
Glyma.13G171400.1(GmXAP4)       LKWTPIQPIAEEG--------------GQRGTQRLSPGGPDAHHH----
AT4G18510.1(CLE2)               TSPTVEDDQAAGS--------------HGKSPERLSPGGPDPQHH----
Potri.019G091100.1              SKQLFEDDSDVTG--------------SPYEPKRISPGGPDPKHH----
LOC_Os01g55080.1                MKVPAAAAGEAAGRRSGGGAVVVVGGAAAHESKRLSPGGPDPQHH----
                                 .          .                  .:*:******.:**
```

Figure S4. Amino acid sequence alignment of the precursor polypeptides of tomato xylem exudate-associated peptides and their homologs in various plants. The predicted signal peptides are underlined (SignalP 3.0 https://services.healthtech.dtu.dk/service.php?SignalP-3.0). The mature peptide regions are shown in red.
